# Negative Life Events in Childhood as Risk Indicators of Labour Market Participation in Young Adulthood: A Prospective Birth Cohort Study

**DOI:** 10.1371/journal.pone.0075860

**Published:** 2013-09-11

**Authors:** Thomas Lund, Johan Hviid Andersen, Trine Nøhr Winding, Karin Biering, Merete Labriola

**Affiliations:** 1 Danish Ramazzini Centre, Department of Occupational Medicine, Regional Hospital, Herning, Herning, Denmark; 2 National Centre for Occupational Rehabilitation, Rauland, Norway; 3 Department of Clinical Social Medicine, Public Health and Quality Management, Central Denmark Region and Section of Clinical Social Medicine and Rehabilitation, School of Public Health, University of Aarhus, Aarhus, Denmark; Hunter College, City University of New York (CUNY), CUNY School of Public Health, United States of America

## Abstract

**Background:**

Most previous studies on reliance on social benefits have focused on health, sickness absence, work environment and socioeconomic status in adulthood. Extending the focus to include early life circumstances may improve our understanding of processes leading to educational and occupational marginalisation and exclusion. The aim of this study was to investigate if multiple negative life events in childhood determined future labour market participation, and to identify important negative life events for labour market participation in young adulthood.

**Methods:**

Of a cohort of 3,681 born in 1989 in the county of Ringkjoebing, Denmark, 3,058 (83%) completed a questionnaire in 2004. They were followed in a register on social benefits for 12 months in 2010-2011. Logistic regression analyses were used to investigate associations between negative life events in childhood and future labour market participation, taking into account effects of socio-economic position, school performance, educational plans, vocational expectations and general health.

**Results:**

A total of 17.1% (19.9% males, 14.4% females) received social benefits for at least 4 weeks during follow-up. Labour market participation decreased with number of negative life events, especially for females: Females who had experienced their parents’ divorce, had been abused, or had witnessed a violent event, showed decreased labour market participation, when adjusting for SES, school performance, educational plans, vocational expectations and general health at baseline. Attributable fractions ranged from 2.4% (parents’ alcohol/drug abuse) to 16.1% (parents’ divorce) for women. For men, risk estimates were lower and insignificant in the most adjusted models. Attributable fractions ranged from 1.0% (parents’ alcohol/drug abuse) to 4.9% for witnessing a violent event.

**Conclusions:**

Information on childhood conditions may increase the understanding of determinants of labour market participation for young adults. Knowledge of negative life events in childhood should be taken into account when considering determinants of labour market participation and identifying high-risk groups.

## Introduction

Early marginalisation from working life among young people has caused growing concerns in several industrialised countries [1]. Understanding the determinants leading young people to dependency of social benefits rather than starting an education or entering working life is therefore of great interest for both researchers and decision makers, especially in times of economical decline. It is generally accepted, that education improves job prospects in general and the likelihood of remaining employed in times of economic hardship [2]. Also, positive early work experiences and education are critical to development of young people into healthy adults [3]. The risk of being excluded from the educational system and the labour market has shown to have negative health consequences, and during a severe economic recession, entry into working life for younger people becomes harder to achieve [1]. OECD finds that the worsening conditions on the OECD labour market are more severe for younger workers than for older workers. On the other hand, it could be argued that the ageing of the population and the decline in the population of 15-29 year-olds in OECD countries favour employment among young adults [1]. Nevertheless, in order to ensure future demands of labour and decrease the individual and societal costs associated with poor labour market participation, it is important to understand the mechanisms behind not entering into education or work in the early years of life, and rather rely on social benefits. Previous studies have concentrated on adulthood predictors of receiving social benefits, such as health status, sickness absence, work environment and socioeconomic status (SES) [4–6]. Extending the focus to cover potential determinants from earlier life circumstances seems to be justified: A Finnish longitudinal cohort study found a 3.5-fold risk of disability pension when the respondents had experienced 5–6 negative events (divorce or separation of parents, long-term financial difficulties in the family, frequent fear of a family member, severe illness of a family member and alcohol-related problems of a family member) during childhood [7]. A British birth cohort study on life course approach to long-term sickness absence, found that a measure of IQ, at age 11, had an impact on long-term sickness absence measured at age 42 [8]. Additionally, experience of stressful life events is a well-established risk factor for health measures such as the development of depressive problems in adolescence and adulthood [9–15]. However, expanding focus to include determinants from early life may increase our understanding of the complex process and determinants non-participation in working life, and provide insight into the mechanisms that determine why working life for some is over before it has begun.

The purpose of this study was to investigate whether adverse circumstances in terms of specific negative life events (NLE) in childhood determined later labour market participation, and to identify which negative life events constituted the largest risk. The analyses took into account the simultaneous effects of socio-economic position, school grades, educational plans, vocational expectations and general health at age 14-15.

## Methods

### Ethics Statement

The study was approved by the Danish Data Protection Agency, according to Danish law for studies using questionnaire and register data (The Act on Processing of Personal Data - Act No. 429 of 31 May 2000). Informed consent was not required as data were analyzed anonymously.

### Population

The source population for the present study consisted of individuals born in 1989 in the county of Ringkjoebing, Denmark and still living there in early April 2004. A total of 3,681 fulfilled these criteria, and contact information was retrieved from the Central Office of Civil Registration and from public schools in the county of Ringkjoebing. All 3,681 individuals were contacted and asked to fill out a questionnaire. Recruitment took place at the schools within the county where a baseline questionnaire was filled out during school hours. Those not at school on the day of collection received the questionnaire by mail. A total of 3,058 participated in the questionnaire survey, corresponding to a response rate of 83%.

### Data

Information for the present study derived partly from a questionnaire survey conducted in spring 2004, when the respondents were 14-15 years old, and partly from register data on social benefits gathered from September 2010 to September 2011.

Data on social benefits derived from the DREAM register [16,17], a national register on all transfer payments from mid 1991 until September 2011 at the time of this study. DREAM includes payments related to unemployment benefits, sickness absence compensation, disability pension, state educational grants, immigration and death, and was merged with the questionnaire data using the CPR number of each participating individual.

### Outcome

The outcome of this study, labour market participation (LMP), was defined according to degree of receiving social benefits in a 52-week period from week 35 in 2010 through week 34 in 2011, when the respondents were 21-22 years old. People were divided into two LMP categories, depending on receiving social benefits: Those not receiving any social benefits, receiving maternity leave benefits or state educational grants, and those receiving all other social benefits. These benefits were either health related benefits (sickness absence compensation, vocational rehabilitation benefits, permanent disability benefits) or unemployment benefits of some sort, including leave benefits and training benefits offered to people after a 4 weeks of unemployment. The two LMP categories were further defined as either “High” (those who did not receive any benefits, received maternity leave benefits or state educational grant for at least 48 of the 52 week follow-up period) and “Low” (those who were on any other kind of social benefit other than maternity leave and State Educational Grant for more than four of the 52 week follow-up period).

### Risk factors

#### Negative life events

Negative life events (NLE) were assessed using 6 items, partly from a 13 items scale developed by Newcomb, Huba and Bentler [18] and items from The Social Stress Indicator developed by Turner, Wheaton and Lloyd [19]. The six questions were: “In your life-time….”: 1: “Have your parents divorced?” 2: “Have you lost any of your parents because they died?” 3: “Have any of your parents abused alcohol or drugs to an extent where it caused problems in the family?” 4: “Have you been abused by someone you knew?” 5: “Have you witnessed a very violent event?” and 6: “Have your parents suffered a life-threatening disease or accident?”. Response options were yes/no. A missing answer was classified as not having experienced the adverse childhood circumstance, given that the respondent had replied to at least one of the other specific questions. NLEs were subsequently divided into 3 categories according to number of NLEs: 0, 1-2, and 3-5, inspired by Harkonmäki and colleagues 2007 [7].

#### Socio-economic position

Socio-economic position was measured using the MacArthur Scale of Subjective Social Status [20], asking respondents to place an “X” of where they stand on a ladder depicting the respondents perceived social level in the Danish society. The ladder has ten steps, which are subsequently coded into an SES variable ranging from 1–10.

#### School grades

School grades were assessed with two questions on self-reported grades in the latest test in Danish and mathematics. At the time of the questionnaire survey, grades were awarded on a 10-point scale ranging from 0 to 13 in the order: 0, 3, 5, 6, 7, 8, 9, 10, 11, and 13.

#### Educational plans and vocational expectations

Educational plans were measured with a question on whether or not the respondent had firm educational plans for the next 2 years, with six response options. Five of these reflected various firm scenarios such as continuing in high school or similar, whereas one option was “I don’t know”. Responses were dichotomized into those with firm plans vs. those who answered “I don’t know”.

Vocational expectations was assessed with a similar tool, where respondents were asked in an open question to specify which type of job they imagined having at the age of 30. There was also an option to tick off the response option “I don’t know”, and responses were dichotomized into those specifying a job and those who used the “I don’t know” option.

#### Health

Global self-rated health (SRH) was measured using a single question: “How do you rate your health in general?” with five response options (very good, good, fair, poor, very poor) [21].

#### Background variables

The data furthermore contained information on the gender of the respondents.

### Analysis

Initial analysis of univariate associations between various risk factors and LMP was performed using Chi Square test and Students T-test, depending on type of variable. Logistic regression methods were used to analyze the association between NLE measured at baseline and LMP at follow-up. Analysis was performed for three exposure categories (0, 1-2, 3-5) according to numbers of NLE during childhood, as well as for each single NLE item. These analyses were performed in four steps: Step one presented the crude OR for associations, step two was adjusted for SES, step three was further adjusted for school grades, educational plans and vocational expectations, and the final step four was further adjusted for self-rated health. In step 1 we tested for interaction between NLEs and LMP for men and woman - no significant interactions were found. Attributably fraction (AF) was calculated for each single NLE item, based on the most adjusted estimate for the effect of the NLE item on LMP [22]. All logistic regression analyses were stratified by gender. All analyses were performed using STATA MP2 version 12 for Mac OS X. [23]

## Results

A total of 17.1% were categorized with low labour market participation at follow-up, significantly more men than women (Table 1). Across gender, a total of 78.2% did not receive social benefits at any time during the 12-month follow-up period, whereas 10.8% received benefits up to 25% of the 12-month period, an additional 4.2 received benefits from 25% to 50% of the period, further 2.4% from 50% to 75% and finally 4.3% from 75% to 100%. Of the latter group, a total of 2.7% received social benefits for the full 12-month follow-up period. Looking at the combined bulk of social benefits during the 12-month follow-up period, the majority of benefits (80%) fell within unemployment related benefits, whereas as 20% were health related (sickness absence compensation). Analysing the use of social benefits from age of eligibility (18 years), showed that 9 individuals (0.3%) were on permanent disability pension, 18.3% had at some point received sickness absence compensation, whereas 11% had at some point received unemployment benefits (not shown). Unpublished analyses have confirmed that responders and non-responders do not differ on educational and vocational outcomes (Winding TN, personnel communication).

**Table 1:** Labour Market Participation (LMP) at age 21-22 in 2010-2011 distributed by gender.

Variable	Level	Women, N (%)	Men, N (%)	Total, N (%)	P, gender diff.
LMP	High	1,317 (85.6)	1,217 (80.1)	2,534 (82.9)	<0.000
	Low	221 (14.4)	303 (19.9)	524 (17.1)	

N=3,058.

The MacArthur Scale score of self-assessed SES strata in childhood was only marginally associated with future LMP. The number of childhood adversities experienced before age 14-15 was significantly associated with future LMP, and especially parents’ divorce, parents’ alcohol/drug abuse, having been abused or having witnessed a violent event, was associated with low labour market participation. Having fixed educational plans at the age of 14-15 and firm vocational expectations were not associated with future LMP, whereas school grades were. Self-rated health at age 14-15 was not associated with later LMP (Table 2).

**Table 2:** Risk indicators at age 14-15 in 2004 of Labour Market Participation (LMP) at age 21-22 in 2010-2011.

Risk factor	Level	High LMP	Low LMP	Total	P, LMP diff.
		N (%)	N (%)	N (%)	
Gender	Women	1,317 (52.0)	221 (42.2)	1,538 (50.3)	<0.000
	Men	1,217 (48.0)	303 (57.8)	1,520 (49.7)	
SES	Highest tertile	831 (33.7)	156 (30.5)	987 (33.2)	0.061
	Mid tertile	714 (29.0)	136 (26.6)	850 (28.6)	
	Lowest tertile	918 (37.3)	219 (42.9)	1,137 (38.2)	
Negative life events	0	1,550 (62.3)	268 (52.8)	1,818 (60.7)	<0.000
	1-2	842 (33.8)	200 (39.4)	1,042 (34.8)	
	3-5	96 (3.9)	40 (7.9)	136 (4.5)	
Parents divorced	No	1,988 (79.2)	383 (74.4)	2,371 (78.4)	0.016
	Yes	523 (20.8)	132 (25.6)	655 (21.6)	
Lost parents	No	2,438 (97.1)	496 (96.1)	2,934 (96.9)	0.245
	Yes	73 (2.9)	20 (3.9)	93 (3.1)	
Parents alcohol/drug abuse	No	2,353 (93.8)	461 (89.7)	2.814 (93.1)	0.001
	Yes	155 (6.2)	53 (10.3)	208 (6.9)	
Abused	No	2,460 (98.0)	479 (93.0)	2,939 (97.2)	<0.000
	Yes	50 (2.0)	36 (7.0)	86 (2.8)	
Witness to violent event	No	2,289 (91.3)	438 (85.4)	2,727 (90.3)	<0.000
	Yes	218 (8.7)	75 (14.6)	293 (9.7)	
Parents accident/disease	No	2,122 (84.7)	425 (83.0)	2,547 (84.5)	0.323
	Yes	382 (15.3)	87 (17.0)	469 (15.5)	
Educational plans	Yes	2,297 (92.6)	463 (91.5)	2,760 (92.4)	0.420
	No	185 (7.4)	43 (8.5)	228 (7.6)	
Voc. expectations at 30 yrs	Yes	1,552 (61.3)	323 (61.6)	1,875 (61.3)	0.866
	No	982 (38.8)	201 (38.4)	1,183 (38.7)	
Danish grades	Mean (range 0-13)	8.6	8.3	8.6	<0.000
Math grades	Mean (range 0-13)	8.6	8.1	8.6	<0.000
Self-rated health	Good	2,413 (95.9)	495 (95.0)	2,908 (95.7)	0.378
	Poor	104 (4.1)	26 (5.0)	130 (4.3)	

N=3,058.

The number of NLE was significantly associated with future LMP among females in a dose–response manner: The unadjusted ORs for having experienced 1-2 or 3-5 NLE vs. 0 were 1.54 (95% CI 1.13-2.11) and 3.50 (2.09-5.85) respectively. This only altered slightly when taking into account the simultaneous effects of SES, school grades, educational plans, vocational expectations and self-rated health, yielding ORs of 1-2 or 3-5 NLE of 1.54 (95% CI 1.07-2.22) and 3.37 (95% CI 1.82-6.24) respectively in the most adjusted model (Table 3, model IV). The risk associated with childhood adversities was generally smaller for males, and became insignificant when entering school grades and educational plans and vocational expectations into the model (Table 3). For men, test for trend was significant for models 1 and 2 (p=0.01 and 0.05 respectively), but insignificant in models 3 and 4. For women, test for trend was significant in all models (not shown).

**Table 3:** Risk of low labour market participation among women (n=1,538) and men (n=1,520) associated with number of negative life events (NLE) in childhood.

			Model I	Model II	Model III	Model IV
	NLE	N				
Women	0	879	1 (-)	1 (-)	1 (-)	1 (-)
	1-2	536	1.54 (1.13-2.11)	1.53 (1.12-2.09)	1.58 (1.10-2.27)	1.54 (1.07-2-22)
	3-5	82	3.50 (2.09-5.85)	3.54 (2.11-5.95)	3.52 (1.91-6.48)	3.37 (1.82-6.24)
Men	0	939	1 (-)	1 (-)	1 (-)	1 (-)
	1-2	506	1.30 (1.00-1.70)	1.27 (0.97-1.67)	1.13 (0.82-1.56)	1.11 (0.81-1.55)
	3-5	54	1.74 (0.94-3.23)	1.51 (0.80-2.87)	0.96 (0.38-2.41)	0.99 (0.39-2.49)

OR and 95% CI.

Model I crude. Model II adjusted for SES. Model III further adjusted for educational factors. Model IV further adjusted for SRH

Among females, separate analysis of the individual NLE items yielded a significant effect of experiencing ones’ parents divorcing, having parents who abused alcohol or drugs, being abused by someone known to the respondent, and having witnessed a violent event. These effects persisted independently of SES score. The effect of parents’ alcohol/drug abuse became insignificant when entering the four school variables into the model, leaving parents’ divorce (OR 1.82, 95% CI 1.26-2.63), being abused (OR 5.10, 95% CI 2.60-9.97) and having witnessed a violent event (OR 2.47, 95% CI 1.54-3.94) as significant risk indicators in the most adjusted models (Table 4).

**Table 4:** Risk of low labour market participation among women (n=1,538) and men (n=1,520) associated with different negative life events (NLE) in childhood.

	Negative life event	Model I	Model II	Model III	Model IV	
						AF
Women	Parents divorced	1.84 (1.35-2.52)	1.84 (1.34-2.52)	1.86 (1.29-2.68)	1.82 (1.26-2.63)	16.1%
	Lost parents	1.73 (0.87-3.44)	2.00 (0.99-4.01)	1.92 (0.84-4.40)	1.87 (0.82-4.28)	2.8%
	Parents alcohol/drug abuse	1.71 (1.08-2.71)	1.72 (1.08-2.73)	1.37 (0.77-2.42)	1.31 (0.74-2.33)	2.4%
	Abused	4.89 (2.79-8.57)	4.92 (2.79-8.65)	5.40 (2.78-10.49)	5.10 (2.60-9.97)	12.7%
	Witness violent event	2.21 (1.46-3.33)	2.23 (1.47-3.37)	2.56 (1.61-4.08)	2.47 (1.54-3.94)	12.0%
	Parents acc./disease	1.09 (0.75-1.58)	1.06 (0.73-1.56)	1.26 (0.83-1.92)	1.21 (0.79-1.85)	3,5%
Men	Parents divorced	0.99 (0.72-1.36)	0.97 (0.70-1.34)	0.80 (0.53-1.21)	0.79 (0.52-1.20)	-
	Lost parents	1.07 (0.51-2.25)	1.02 (0.48-2.16)	0.65 (0.19-2.20)	0.66 (0.19-2.24)	-
	Parents alcohol/drug abuse	1.96 (1.22-3.16)	1.78 (1.09-2.91)	1.17 (0.61-2.27)	1.18 (0.61-2.29)	1.0%
	Abused	2.84 (1.39-5.81)	2.74 (1.33-5.63)	1.72 (0.68-4.33)	1.75 (0.69-4.41)	1.6%
	Witness violent event	1.51 (1.02-2.21)	1.52 (1.03-2.23)	1.53 (0.98-2.40)	1.51 (0.95-2.38)	4.9%
	Parents acc./disease	1.25 (0.88-1.77)	1.14 (0.79-1.63)	0.92 (0.59-1.44)	0.96 (0.61-1.50)	-

OR and 95% CI for response option “yes” compared to “no”.

Model I crude. Model II adjusted for SES. Model III further adjusted for educational factors. Model IV further adjusted for SRH

For males, the crude models yielded a significant effect of having parents who abused alcohol/drugs, having been abused, and having witnessed a violent event. The associations became insignificant when entering the four variables assessing school grades, educational plans and vocational expectation into the model, yielding no significant associations of childhood adversities on future LMP among males.

Attributable fractions (AF) were calculated for childhood adversities based on the most adjusted models. For women, AFs ranged from 2.4% for parents alcohol/drug abuse, to 16.1% for parents divorce. Being abused in childhood attributed 12.7% of the excess risk of future low labour market participation. For males, NLE explained less than was the case for women: AFs ranged from 1.0% (parents abused alcohol/drugs) to 4.9% for those who witnessed a violent event.

## Discussion

The present study showed, that among a cohort of 14-15 year olds, 17.1% received social benefits for at least 4 weeks in a 12-month period seven years later at the age of 21-22 years. This was the case for significantly more males (19.9%) than females (14.4%). The risk of low labour market participation increased if one had experienced certain negative life events in childhood, especially for females. Females who in their childhood had experienced their parents’ divorce, had been abused, or had witnessed a very violent event, had increased risk of future low labour market participation, when adjusting for SES, school performance, educational plans, vocational expectations and general health at age 14-15. Especially those who had been abused by someone they knew had highly increased risk (OR 5.10, 95% CI 2.60-9.97). This findings supports the findings by Harkonmäki and colleagues, who found frequent fear of a family member to be the strongest predictor of disability pension in a similar battery of NLEs in childhood [7].

Attributable fractions of various NLE ranged from 2.4% (parents’ alcohol/drug abuse) to parents divorce (16.1%) for women. For men, risk estimates were generally lower and tested statistically insignificant in the most adjusted models. Attributable fractions ranged from 1.0% (parents’ alcohol/drug abuse) to 4.9% for witnessing a very violent event.

When interpreting the study findings, one should consider the potential error induced by the 7 years from baseline to follow-up, where we have no information about the study participants. Life events could occur that affect LMP, which were unmeasured in this study. An individual experiencing for example an NLE during follow-up, would appear in the group of unexposed in the performed analyses. This could potentially underestimate the effects of NLEs on LMP. Regarding the measurement of NLE, this covers events taking place in an age span of 0-14/15 years of age, and therefore covers both early childhood, middle childhood and adolescence. Asking about events in early childhood could potentially introduce recall bias. This could potentially underestimate the effects of NLEs on LMP. However, considering the severity of the NLEs assessed in this study, we consider recall bias to be a minor issue. Another concern is the subjective nature inherent in certain of the NLE items. Surely, whether or not ones parents have been divorced or have died is not open for interpretation, but, for example, the degree to which an event is “very violent” or not, is to a large extent a matter of personal norms. Unpublished analyses of the data showed, that females more often report to have witnessed a very violent event than males did, but whether or not this is due to females witnessing more violence or interpreting violent episodes differently than males do, is unknown. However, in relation to this study, we argue that it is the experience that constitutes the adversity, rather than, in the case of this example, how one assesses a specific level of violence.

On the positive side, the study features a relatively high participation rate of 83%. An additional strength is the 100% follow-up due to use of register data, which furthermore eliminates common method variance [24]. A further advantage compared to the study by Harkonmäki and colleagues [7] is this study’s assessment of adverse circumstances in childhood while the study participants are still children, which minimizes potential recall bias compared to asking adults about events in early childhood.

The present study does not shed light on the pathway from NLE to future LMP: It can be hypothesised that NLE affects health [9–15,25], which again can affect LMP. To which degree this is the case for the participants in this study is unknown, but the larger proportion of the social transfer payments defining “low LMP” were related to unemployment benefits rather than health related benefits. We adjusted for health in Model 4 only, as the role of health as a confounder is dubious. Previous studies have also shown a strong association between socio-economic status and not only health [1,3,26–28], but also labour market attachment [4–6]. The present study only found a borderline significant association between socio-economic position in childhood and LMP in young adulthood. According to Rahkonen et al., both past and present socioeconomic status are important determinants of adult health [27]. This discrepancy could be due to the assessment of SES in this study: Strong evidence of the construct validity of the MacArthur scales is found in both middle-aged and older men and women [28], and the validity in relation to young people has been tested with good results previously [29–31]. However, Rahkonen et al. argue that the socioeconomic status of destination, that is one’s own education, has a higher impact on health status across age groups [27]. The weak association between childhood SES and LMP in young adulthood could also be due to the relatively young age of the study participants at follow-up: The differences in labour market participation caused by socio-economic inequalities in childhood might not have come through fully in the early twenties, especially not in Denmark, where people generally are older before entering working life compared to many other countries.

## Conclusion

The effects of negative life events in childhood should be taken into account when considering both the determinants of labour market participation, as well as when identifying high-risk populations. Recognition and alleviation of negative life events in childhood should be promoted in relation to policies and practices aiming at integrating youth, especially young females, into education and working life.
